# The cerebrospinal fluid proteome in HIV infection: change associated with disease severity

**DOI:** 10.1186/1559-0275-9-3

**Published:** 2012-03-20

**Authors:** Thomas E Angel, Jon M Jacobs, Serena S Spudich, Marina A Gritsenko, Dietmar Fuchs, Teri Liegler, Henrik Zetterberg, David G Camp, Richard W Price, Richard D Smith

**Affiliations:** 1 Biological Sciences Division, Pacific Northwest National Laboratory, Richland, WA, USA; 2 University of California San Francisco, San Francisco, CA, USA; 3 Biocenter, Innsbruck Medical University, Innsbruck, Austria; 4 Institute of Neuroscience and Physiology, Department of Psychiatry and Neurochemistry, the Sahlgrenska Academy at the University of Gothenburg, Gothenburg, Sweden; 5 Yale University School of Medicine, New Haven, CT, USA

**Keywords:** Amyloid, Cerebrospinal fluid, HIV, Pathway, Proteomics

## Abstract

**Background:**

Central nervous system (CNS) infection is a nearly universal feature of untreated systemic HIV infection with a clinical spectrum that ranges from chronic asymptomatic infection to severe cognitive and motor dysfunction. Analysis of cerebrospinal fluid (CSF) has played an important part in defining the character of this evolving infection and response to treatment. To further characterize CNS HIV infection and its effects, we applied advanced high-throughput proteomic methods to CSF to identify novel proteins and their changes with disease progression and treatment.

**Results:**

After establishing an *accurate mass and time *(AMT) tag database containing 23,141 AMT tags for CSF peptides, we analyzed 91 CSF samples by LC-MS from 12 HIV-uninfected and 14 HIV-infected subjects studied in the context of initiation of antiretroviral therapy and correlated abundances of identified proteins a) within and between subjects, b) with all other proteins across the entire sample set, and c) with "external" CSF biomarkers of infection (HIV RNA), immune activation (neopterin) and neural injury (neurofilament light chain protein, NFL). We identified a mean of 2,333 +/- 328 (SD) peptides covering 307 +/-16 proteins in the 91 CSF sample set. Protein abundances differed both between and within subjects sampled at different time points and readily separated those with and without HIV infection. Proteins also showed inter-correlations across the sample set that were associated with biologically relevant dynamic processes. One-hundred and fifty proteins showed correlations with the external biomarkers. For example, using a threshold of cross correlation coefficient (Pearson's) ≤ -0.3 and ≥0.3 for potentially meaningful relationships, a total of 99 proteins correlated with CSF neopterin (43 negative and 56 positive correlations) and related principally to neuronal plasticity and survival and to innate immunity. Pathway analysis defined several networks connecting the identified proteins, including one with amyloid precursor protein as a central node.

**Conclusions:**

Advanced CSF proteomic analysis enabled the identification of an array of novel protein changes across the spectrum of CNS HIV infection and disease. This initial analysis clearly demonstrated the value of contemporary state-of-the-art proteomic CSF analysis as a discovery tool in HIV infection with likely similar application to other neurological inflammatory and degenerative diseases.

## Background

While central nervous system (CNS) HIV infection is a nearly universal facet of systemic infection, its course and clinical manifestations are highly variable and, indeed, evolve over time within individual patients. CNS infection can be detected soon after primary exposure and initial viremia [1,2], and continues throughout the chronic course of untreated infection [3-6]. This course has been defined to a great extent by studies of cerebrospinal fluid (CSF), which provides a useful window into CNS infection and disease. CSF analysis has shown that the clinical manifestations of chronic CNS infection range from common asymptomatic infection, often accompanied by clinically silent CSF pleocytosis [5], to more severe "invasive" HIV encephalitis (HIVE) presenting clinically as the AIDS dementia complex (ADC) [7,8], now most commonly referred to as HIV-associated dementia (HAD) [9]. While the factors contributing to this evolution and clinical variability are partially characterized and involve changes in both infecting virus populations and host immune responses, the critical interactions of these two factors that eventuate in brain injury are still not well understood [10,11]. While combination antiretroviral therapy (cART) has been very effective in reducing the incidence of severe HAD [12,13], milder neurological impairment is noted in otherwise well-treated populations [14-16] and is now of particular concern as surviving patients age [17].

Because of its close proximity to the brain parenchyma and interchange with brain extracellular fluid, the composition of CSF can provide insights into the brain chemistry and cellular processes of infection and brain perturbation, and because it is also separated from the blood by barriers that restrict molecular and cellular passage, it can also serve as a "model" of an isolated and protected tissue reacting to infection that shares properties with brain [18,19]. These characteristics, along with the safety of CSF sampling - including serial sampling for longitudinal observation - have provided an informative view of CNS HIV infection across the spectrum of systemic and CNS disease and the effects of treatment. CSF studies have shown the evolution of compartmentalized CNS viral populations [20-22], CNS immune and inflammatory reactions [23-25] and CNS injury [26,27] from primary infection to advanced HAD/HIVE. CSF studies have also been important in establishing the CNS impact of cART [5,28] and, at times, its shortcomings [29,30]. Previous studies of CSF have involved a variety of defined biomarkers (for review, see Cinque et al. [31]), including most importantly biomarkers related to the three principal components of HIV-related CNS disease: the virus that appears to drive disease, immune responses that may protect but also mediate injury, and, finally, brain cell injury [32]. Despite these previous studies, understanding of infection and brain injury is still incomplete, and there are important gaps in our knowledge of disease evolution and pathogenesis.

In this study, we applied recent advances in high-throughput, mass spectrometry-based proteomic methods to CSF across the spectrum of HIV infection and resultant CNS disease. Proteomic technologies have significantly matured in the last few years, particularly for application in the clinical realm, where robust measurements can now be made with sufficient sensitivity and throughput to allow both the depth and breadth of analysis required for confident global comparisons of larger sample sets [33,34]. Past studies on the CSF proteome of subjects with HIV dementia identified a modest number of proteins differing between control and HIV-infected subjects [35]; however, through utilization of a higher sensitivity and throughput discovery strategy, greater depth of CSF proteome coverage and hence a more comprehensive survey of disease-related changes across the CSF proteome were observed. In recent years, the application of mass spectrometry for the analysis of the CSF proteome has been successfully demonstrated for normal subjects [36] and in neurological diseases [37-41]. Advances in instrumentation, specifically high-efficiency ion transmission technologies [42] with increased detection sensitivity for global proteome studies and the *Accurate Mass and Time *(AMT) tag strategy for high-throughput label-free quantitative analytical measurements of protein abundance [33,43], have facilitated increased depth and breadth of CSF proteome coverage.

We applied proteomic analysis to a group of specimens from well-characterized subjects. Because of anticipated individual between-subject differences unrelated to HIV infection, these initial efforts focused on analysis of longitudinal sample sets, and particularly sample sets for subjects who changed during the period of observation related to either the development of neurological disease or by responses to cART. In addition to analyzing correlations within the proteomic data, we analyzed correlations of identified protein with three orthogonal biomarkers related to main pathobiological disease components. These markers included CSF HIV RNA to assess relations to the local viral replication; CSF neopterin to compare to the state of intrathecal immune activation and particularly macrophage activation [25,44], and CSF neurofilament light chain protein (NFL) as a marker of neuronal-axonal injury [45,46]. Exploration of network and pathway analyses revealed protein interactions potentially involved in CNS injury, and led to the identification of numerous potentially useful markers and pathways for future study.

## Results

### Defining the CSF AMT tag protein database library

Proteomic analysis of CSF involved two stages. The first established an AMT tag database by detailed analysis of four CSF samples subjected to extensive fractionation, allowing for a deep view into the CSF proteome and defining a large peptide-protein library of AMT tags for subsequent application to the larger sample set. Two of the samples were from HIV-uninfected, neurologically normal control volunteers and two from HIV-infected subjects with more advanced infection. The two control subjects, both male ages 45 and 43, had normal CSF WBC counts, total proteins, and albumin ratios along with normal bedside neurological examinations and QNPZ-4 test scores. The two HIV-infected subjects, ages 38 and 45, had advanced systemic infection with blood CD4+ counts of 98 and 84 cells/μL, blood HIV RNA concentrations of 522,000 and 109,000 copies/mL and CSF HIV RNA concentrations of 1,202,000 and 22,600 copies/mL. Their CSF total proteins were elevated at 93 and 60 mg/dL with albumin ratios of 12.79 and 7.63 and CSF cell counts of 35 and 44 WBCs/μL. They also tested abnormally with QNPZ-4 scores of -1.8 and -1.0.

The four samples were subjected to protein isolation, tryptic digestion and pre-MS SCX fractionation. Each SCX fraction was analyzed by high resolution capillary LC-MS/MS for identification of peptide sequences and inclusion into a CSF exclusive AMT tag database. A total 23,141 confident peptide mass and time tag identifications were then included in the CSF database that served as the basis for protein identification in the subsequent main clinical study.

### Characteristics of CSF sample set for the main proteomic analysis

**Figure 1:**
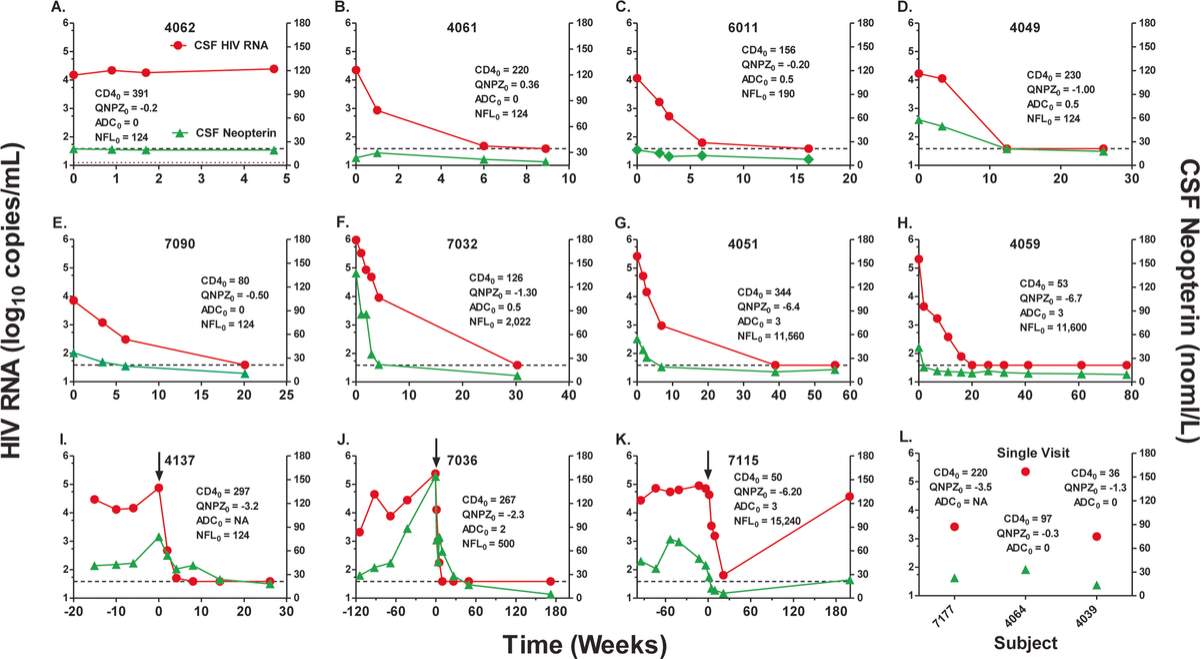
**CSF HIV RNA and neopterin in HIV-infected subjects**. The panels show the longitudinal changes (**A - K**) and the three singly sampled results for CSF HIV RNA and CSF neopterin from the 14 HIV-infected subjects. In the longitudinal panels all subjects initiated cART at time 0 (subjects in panels I-K were observed before starting therapy which is marked by vertical arrow and 0 time scale). The blood CD4+ T cell counts, QNZP-4 performance score, ADC stage and CSF NFL at this baseline are shown in each panel. In the aggregate these sample a broad range of subjects in different stages of untreated and treated HIV infection and CNS disease. Symbols defined in panel A apply to all the panels.

Analysis of the CSF proteome across the spectrum of HIV infection included 79 samples from 14 HIV-infected subjects with varying systemic and CNS disease severity. The longitudinal samples included multiple time points for 11 of the subjects (median 6, range 4-12 samples) (Table 1). The rationale for using multiple longitudinal samples from relatively few individuals in this initial CSF proteomic study aimed to reduce the influence of individual subject variability unrelated to the state of HIV infection. The timing of CSF sampling and responses to cART, as indicated by changing CSF HIV RNA and neopterin levels, are shown in Figure 1. In the 11 longitudinally studied HIV-infected subjects (Figure 1A-K) the median duration of repeated CSF sampling was 30 weeks (range 5-294 weeks). Neurological symptoms, signs, and performance testing also varied longitudinally in some of these subjects. Hence, the samples spanned a broad range of HIV infection, HIV-related CNS disease states, and responses to therapy. The relationship among these three variables across the entire data set is shown in Additional file 1: Figure S1. This allowed both within- and between-subject comparisons. Single CSF samples from three HIV-infected subjects (Figure 1L) and 12 HIV uninfected control subjects, matched for age and gender with infected subjects, were also analyzed. All samples were analyzed in duplicate, and peptides and proteins were identified and quantified employing the AMT tag database.

**Table 1:** Subject and CSF background characteristics

	HIV Infected Baseline	HIV Infected All Samples Median (IQR)*	HIV Negative
**Number**	14	79	12
**Age (mean years, SD)**	46.6 (7.6)	48.0 (7.9)	45.7 (5.1)
**Gender (M/F)**	12/2	59/20	10/2
**Blood T cells (mean cells/μL, SD)**			
**CD4+**	217.1 (155.9)	268.2 (166.7)	902.4 (265.4)
**CD8+**	896.1 (573.0)	933.7 (662.7)	481.9 (243.6)
**HIV-1 RNA (log10 copies/mL)**			
**plasma**	4.29 (3.74 - 5.15)	3.40 (1.72 - 4.76)	NA
**CSF**	4.92 (4.26 - 5.36)	3.85 (1.71 - 4.47)	NA
**CSF WBCs (per μL)**	15 (6.3 - 20.3)	11 (5.0 - 22.0)	1.0 (0.3 - 2.0)
**CSF Neopterin (nMol/L)**	34.8 (22.2 - 48.7)	23.6 (14.0 - 44.3)	4.6 (3.3 - 6.2)
**CSF NFL (ng/L)**	124 (124 - 1846)	235.2 (124.0 - 2460.0)	124 (124 - 124)
**QNPZ-4**	-1.30 (-3.43 - 0.28)	-1.70 (-3.35 - 0.45)	0.00 (-0.85 - 0.46)
**CSF:blood albumin ratio (mean, SD)**	7.946 (3.508)	7.818 (4.074)	5.355 (2.055)

* Unless otherwise designated as mean and SD

Three approaches were then taken in the analysis of the proteomic results across the sample set: characterization of peptide and protein abundances across all subjects, correlation among proteins across sample sets, and correlation of protein abundances with three external CSF biomarkers.

### Peptides and protein identifications and abundance changes across the subjects

**Figure 2:**
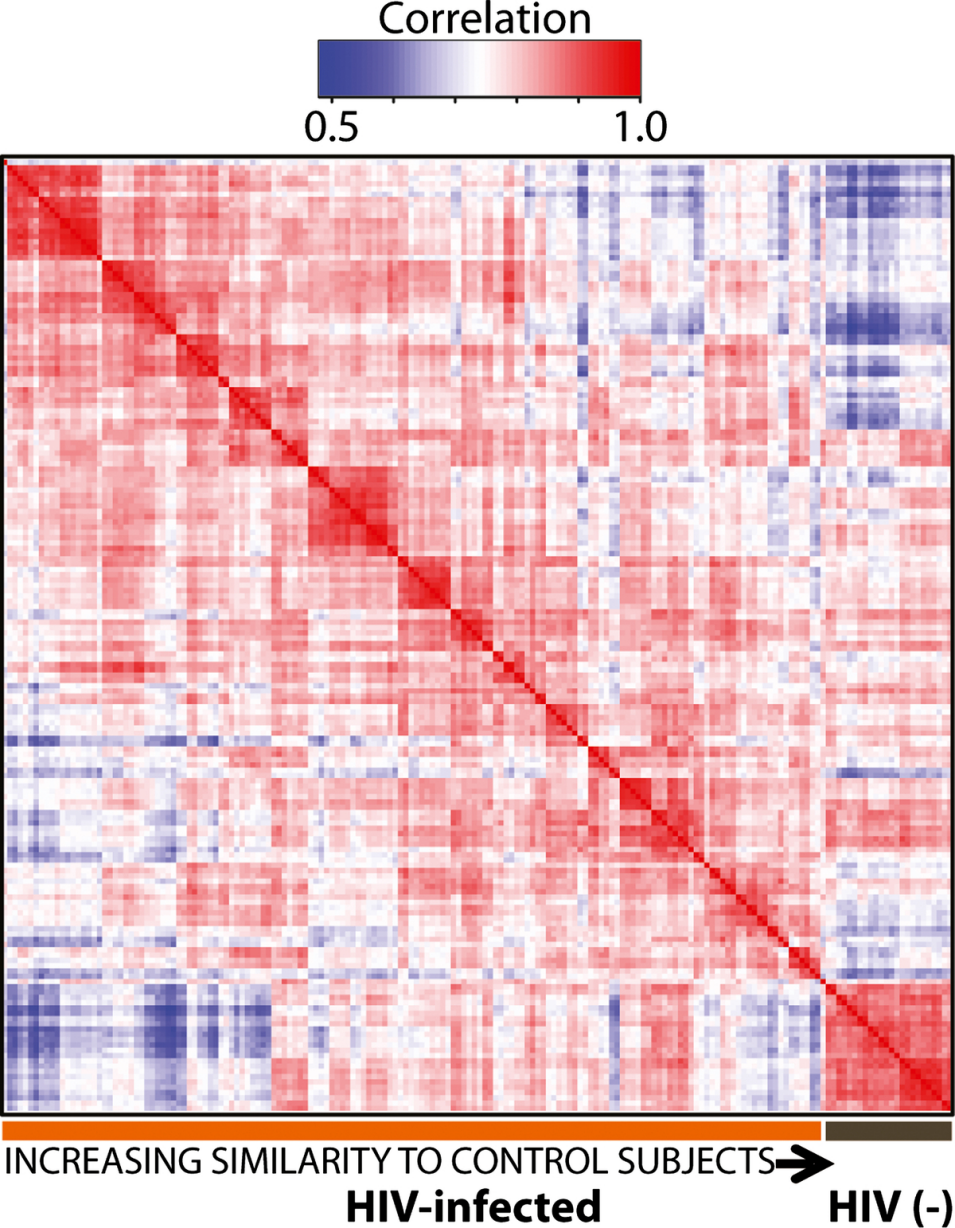
**Hierarchy of protein abundances in across samples**. The CSF proteome profile reveals differences between HIV-infected (+) and HIV-uninfected (-) control samples, demonstrated by unsupervised hierarchical clustering of subjects' CSF proteomes based on Pearson's cross correlation coefficient. Cluster analysis leads to the segregation of HIV (+) and HIV(-) samples, with all HIV negative samples populating the cluster in the lower right corner of the heat map and in serial samples from individuals forming clusters seen as variably-sized red squares in the diagonal. Importantly, these results reveal that the differences between HIV (+) individuals were greater than the differences between serial samples within a single subject.

Analysis of individual CSF samples identified 9,074 peptides (Additional file 2: Table S1) with an average of 2,333 +/-328 (SD) peptides per sample covering 307 +/-16 proteins (Additional file 3: Table S2). Protein identification and quantification were robust, with low levels of sample-to-sample variability in proteins identified. An unsupervised hierarchical cluster analysis of the subject samples, based on the level of Pearson's correlation of protein abundances across individual subject samples clearly discriminated HIV-infected subjects from HIV-uninfected controls, with the latter clustering at the lower right corner of the heatmap representation of these correlations (Figure 2). Sample similarity, as measured by correlation of individual protein abundances, was lower across the HIV-infected samples, with an average cross correlation coefficient of r = 0.77, compared to HIV-uninfected subject samples (r = 0.85). The clustering results revealed that HIV-infected samples also were subdivided and grouped by subject (red squares on the diagonal), indicating that within-subject features transcended many of the between-subject correlations as anticipated. This also resulted in the within-subject correlations being larger (average r = 0.90) than the cross subject correlation (average r = 0.77) for all HIV-infected subject samples.

### Protein correlations across the sample set

**Figure 3:**
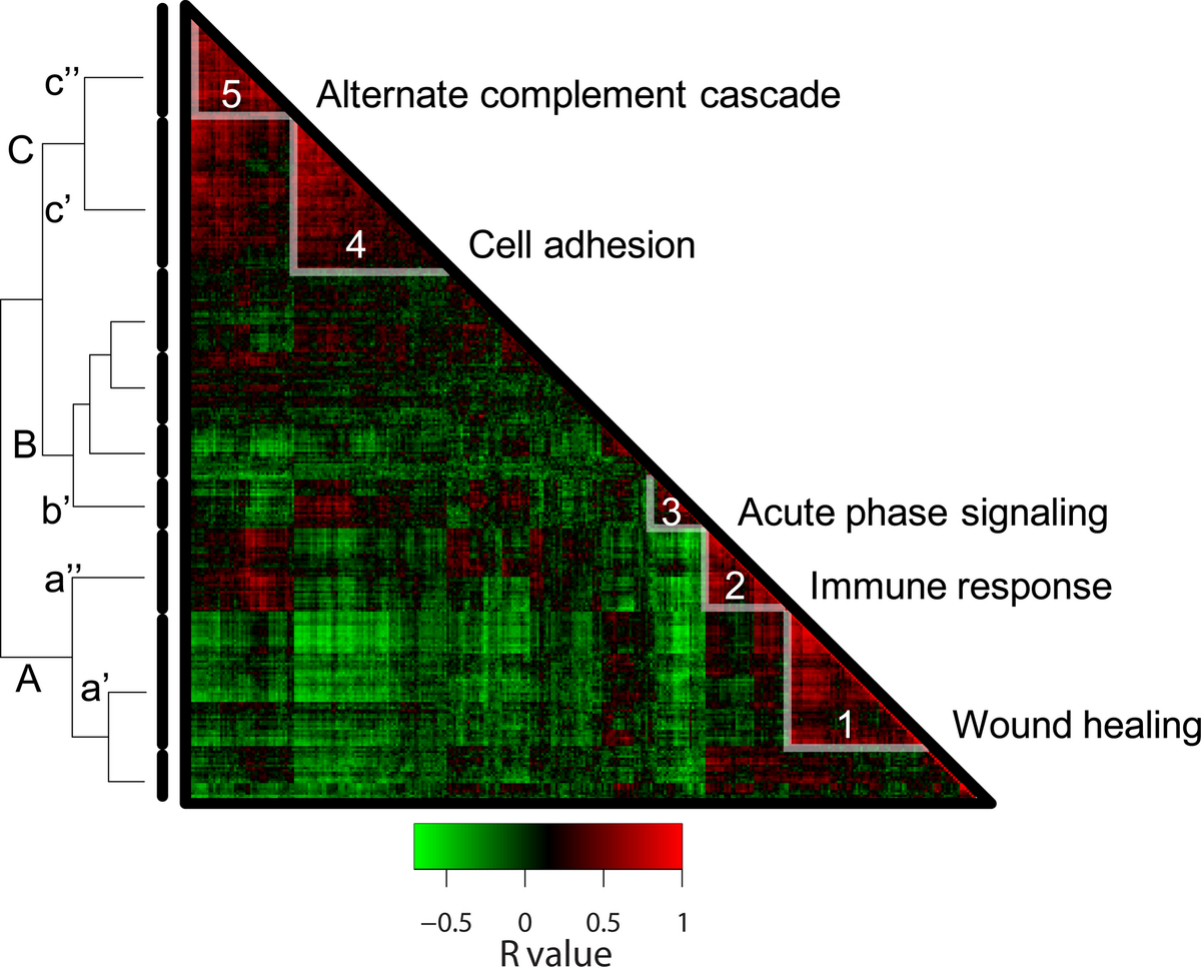
**Hierarchy of protein correlations across sample set**. This depicts the unsupervised hierarchical clustering of proteins identified in the CSF proteome based on protein abundance Pearson's cross correlation coefficient. Protein abundance patterns are either positively correlated (red) or anti-correlated (green). Pathway analysis of clusters revealed that cluster A was composed of proteins that were enriched with respect to functions of wound healing, adaptive immunity, and acute phase signaling. Cluster A could be further separated into two regions depicted as clusters 1 and 2 with strong positive correlation with annotated functional enrichment of wound healing and immune response respectively. Cluster C contains two regions of strong internal correlation with annotated functional enrichment of alternate complement cascade and cell adhesion. Interestingly the proteins in cluster A were largely anti-correlated with proteins in cluster C, and had functional attributes referable to complement and acute phase response as well as cell movement.

Cross sectional analysis of all proteins across the sample set revealed clusters of similarly correlating proteins (Figure 3). The proteins formed three visually prominent clusters annotated in Figure 3 as A-C. Clusters A and C exhibited strong internal positive correlation, but were largely anti-correlated with each other while cluster B (with the exception of group 3) showed lower and more variable levels of correlation within the proteins in this cluster and between clusters A and C. Each cluster could be further broken down into smaller groups exhibiting notable internal correlations warranting closer inspection. Analysis of functional enrichment for members of clusters 1-5 employing GoMiner [47] revealed functional enrichment for proteins that participate in wound healing, immune response, acute phase response signaling, cell adhesion, and the alternative complement cascade, respectively. Notably, cluster A contained groups 1 and 2 that were enriched with proteins relating to wound responses and to blood coagulation (group 1) and to immune and inflammatory response (group 2). Proteins in group 2 show strong positive correlation with group 5, which consisted of proteins with functional enrichment with the alternative complement activation pathway. Group 3, enriched for proteins with functions relating to acute inflammatory response, showed strong positive correlation to group 4, which was enriched for proteins associated with cell adhesion. Thus, overall there was observed a strong cross-protein correlative effect which segregated identified proteins consistent with the inflammatory profile of CSF HIV, in addition to identifying other correlative protein groups across the dataset.

### Protein correlations with 'external' orthogonal CSF biomarkers of HIV infection, local immune activation and CNS injury

**Figure 4:**
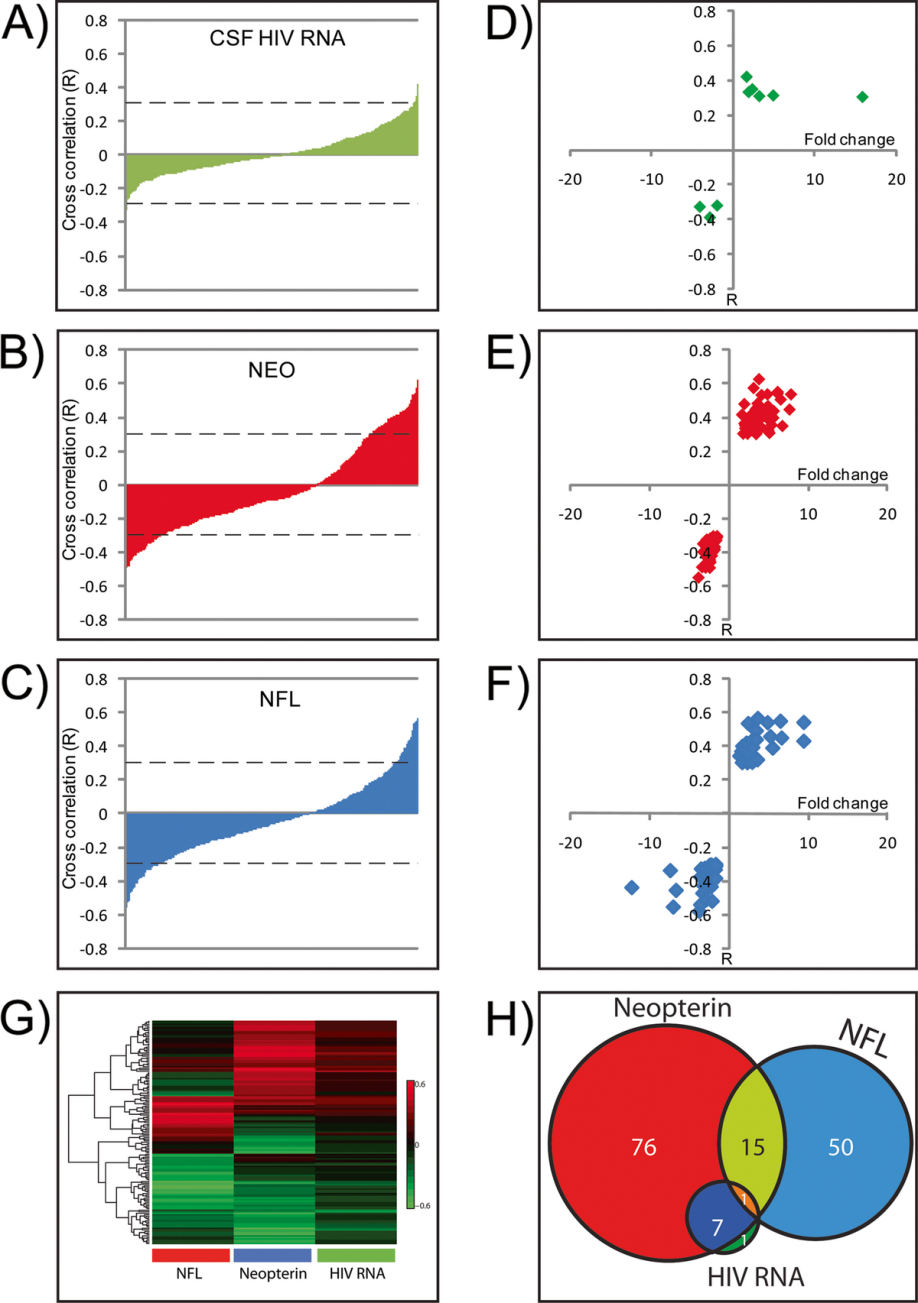
**Cross correlation analysis of proteins quantified in the CSF proteome and orthogonal markers of CNS immune activation (neopterin), neural injury (NFL) and infection (HIV RNA)**. **A-C **plot the distributions of cross correlation coefficients or proteins with these three CSF biomarkers while **D-F **plot the proteins with significant correlations (R ≤ -0.3 or ≥ 0.3 and *p *≤ 0.05) with the respective CSF biomarkers plotted against their respective protein fold changes determined by MS as measured over the course of ART. The heat map (**G**) that includes the cross correlations of proteins correlating with three orthogonal analytical measurements of disease progression shows that distinctly different correlation patterns result from hierarchical clustering of all protein cross correlation values. The Venn diagram (**H**) of proteins that show significant correlations (R ≥ 0.3 or ≤ -0.3, and *p *≤ 0.05) with neopterin (red, n = 99), NFL (blue, n = 66) and CSF HIV RNA (green, n = 9) shows the degree of overlap of correlating proteins.

In parallel with analysis of identified CSF proteins, three constituents of CSF that have served as useful biomarkers of CNS infection and its effects were measured - CSF HIV RNA, neopterin, and CSF NFL as indices of the three principal processes in HIV-related CNS injury: local virus replication, intrathecal immune activation, and neuronal-axonal injury, respectively [32,45,48]. The concentrations of these three biomarkers were then correlated with all individual proteins measured by MS, and those with significant correlations at the defined threshold (*p *≤ 0.05 and r ≥ 0.3 or ≤ -0.3) were selected for further analysis (Figure 4).

In the case of CSF HIV RNA, 9 proteins were identified that met the significance threshold of positive or negative correlation as shown in Figure 4A and 4D and listed in Additional file 3: Table S2). Correlations with CSF neopterin and NFL levels resulted in larger numbers of proteins (99 proteins and 65 proteins, respectively) as shown in Figures 4B, E and 4C, F, and listed in Additional file 3: Table S2. Notably, the three orthogonal measurements, and particularly neopterin and NFL, correlated largely with different groups of proteins as illustrated in Figure 4G and in the related Venn diagram (Figure 4H). This suggests that mostly distinct protein groups are driving or reflecting the pathobiological processes which underlie immune activation (neopterin) and axonal injury (NFL) coincident with HIV infection and disease severity. There were 15 proteins, however, that showed significant correlation with both NFL and neopterin. One of these, clusterin (Gene symbol CLU), was previously reported to be reduced in patients with HIV associated dementia relative to non-demented control samples [35]. Consistent with this, we found clusterin to be anti-correlated with both neopterin and NFL. A single protein was found to correlate with all three markers and it was identified as an antibody Ig kappa V-III chain, likely reflecting adaptive immune activation and immunologic response associated with CNS HIV infection.

### Pathway analysis

**Figure 5:**
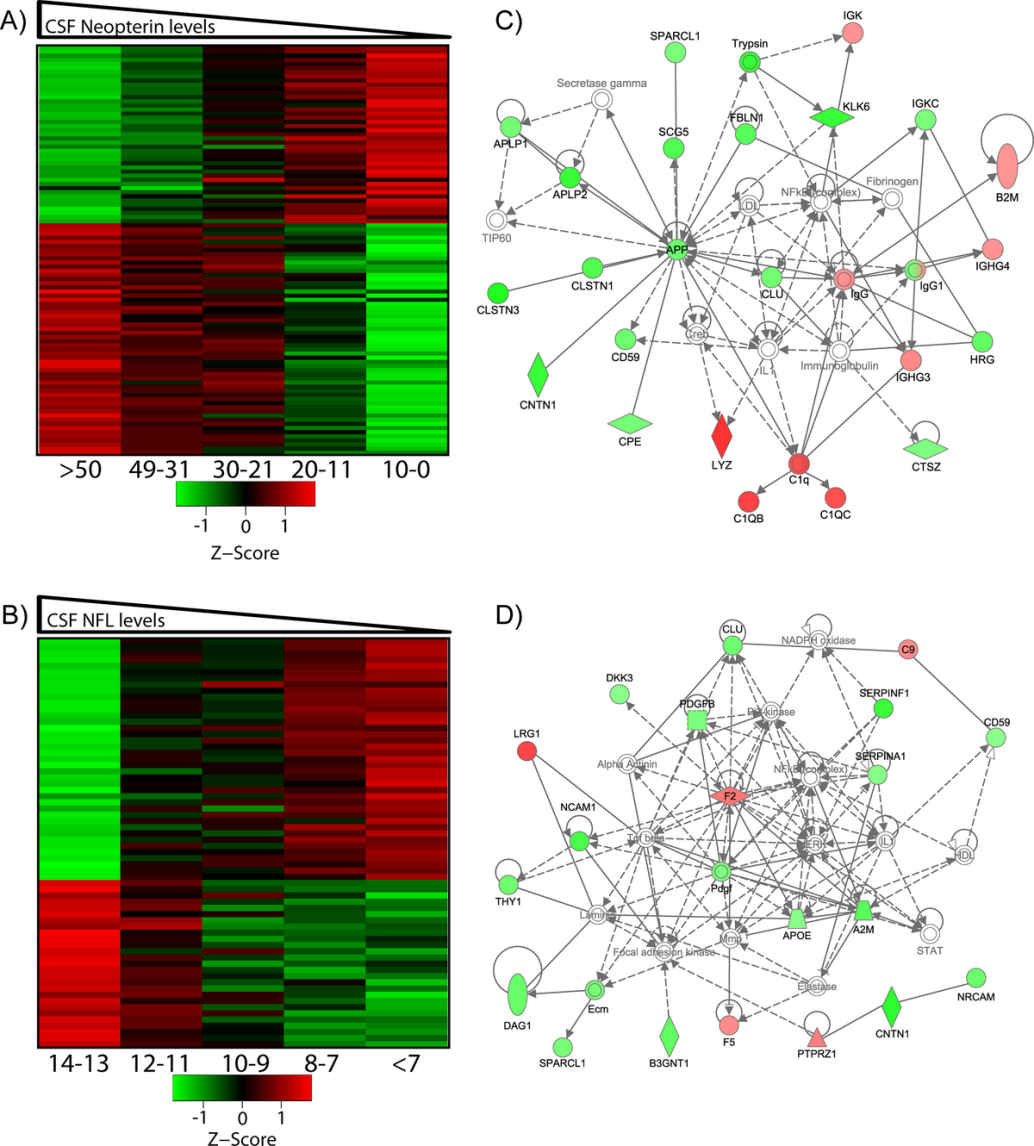
**Heat map showing the changing protein abundances in the CSF as a function of reduction of A) neopterin and B) NFL levels in the CSF**. Average protein abundances measured in CSF samples (grouped by neopterin and NFL values indicated in the heatmap labels) are shown in **A **and **B**. Network analysis of proteins with significant cross correlation with neopterin and NFL resulted in the identification of two distinct networks of proteins with related disease and functional associations shown in **C **and **D**.

Proteins exhibiting strong positive and negative correlations with neopterin and NFL (Figure 5A and 5B) were examined using Ingenuity Pathway Analysis tools http://www.ingenuity.com to annotate associations among the identified proteins and known disease pathways (Figure 5C and 5D). Results suggested that the protein interaction networks related to protein correlates of neopterin and NFL were largely different, with only 4 proteins common to both networks (CD59, clusterin, CNTN1, and SPARKL1). The proteins found in common have annotated functions associated with both immune response regulation (for example, CD59) and normal CNS function. An example of the latter was contactin-1 (gene symbol, CNTN1), a neuronal membrane-associated protein that plays a role in the formation of CNS axonal connections.

Proteins correlating with CSF neopterin levels included amyloid precursor protein (gene symbol, APP), a highly connected node in the network shown in Figure 5C. APP inversely correlated with neopterin, and has been reported to play a role in dampening excitotoxic synaptic stimuli effecting glutamate receptor levels in the synapse [49]. Clusterin (gene symbol, CLU), was linked to APP in the network and also inversely correlated with neopterin. Clusterin is a chaperone similar to the small heat shock proteins and presumably functions as such in the extracellular space in the CSF (reviewed in [50]); it also plays a role in apoptosis-mediated cell death [51].

Proteins correlating with NFL levels in the CSF were used to create the network shown in Figure 5D. This network was different from the neopterin network. Prothrombin (gene symbol, F2), apo-lipoprotein E (gene symbol, APOE), and α2-macroglobulin (gene symbol, A2M) were highly connected nodes within the NFL network. Proteins associated with neuronal development and maintenance, such as neural cell adhesion molecule 1 (gene symbol, NCAM1) and CNTN1 were less centrally connected in the NFL network, though their functions related to axonal damage and maintenance may mean that they are relevant to HIV-related CNS injury.

## Discussion

This exploratory study demonstrates the power of discovery methods applied to CSF in HIV infection and establishes a strong foundation for further extension of these techniques in this condition and to other neurological diseases. In this discovery-phase investigation, MS based label-free quantification was advantageous given no required a priori knowledge of CSF proteome targets. Follow up *validation *studies where targeted approaches are used do require knowledge of target proteins. In searching for disease biomarkers or disease associated protein profiles, the *discovery *phase should provide a list of candidate proteins and serves as a precursor phase for targeted quantitative approaches. These subsequent targeted approaches, whether they use MS techniques or are immunobased, are designed to *validate *the use of the biomarker proteins [52]. The results show both the richness of the CSF proteome and the power of current analytical methods to define its changes in relation to alterations in disease state. Analysis of the CSF proteome allows dissection of protein correlations related to pathogenetic pathways, points to informative CSF proteome biological signatures and identifies potentially useful new biomarkers. The results also demonstrate the value of a strategy using samples derived from longitudinal observations and the sequential analytical approach examining detected proteins: 1. between and within patients; 2. among the identified proteins; and 3. in relation to orthogonal biomarkers using statistical and descriptive pathway analytic approaches.

Clinical and research studies of CSF currently exploit only a narrow range of its content. The improved definition of the CSF proteome presented here indicates considerable potential to expand the range of useful measurements for diagnosis, assessing treatment responses and understanding underlying disease processes in HIV infection. Thus, the study identified a mean of 2,333 +/-328 (SD) peptides per patient sample corresponding to 307 +/-16 proteins and a total of 344 proteins in all CSF samples analyzed by LC-MS (Additional file 3: Table S2), a rich resource to explore for further biomarker definition and insight into the CNS disease pathways. Many (271/344) of the proteins identified in the CSF samples were previously identified in human plasma and reported in the human peptideAtlas database (Additional file 3: Table S2) [53].

### Individual subject proteome signatures

Analysis of protein abundances showed that the majority of samples from the longitudinally-studied individual subjects clustered together in the analysis of overall protein abundances (Figure 2). This was in spite of disease state changes within individual single subjects across time and demonstrates that there are strong individual (*idiosyncratic*) protein "signatures" on which samples can be segregated. This was anticipated in the design of the study, and was the rationale for including longitudinal samples from a relatively small group of subjects with changing clinical state related to cART treatment. While not eliminating the contribution of individual protein profiles, it reduced their impact on the analysis related to changing disease state and treatment effects. It also showed a clear segregation of HIV-infected and -uninfected subject CSF proteome "signatures".

### Correlations among proteins defined by associated abundances

The correlations among proteins across the larger sample set allowed grouping of proteins as they varied in abundance across the disease spectrum, suggesting common or system-level group responses to the pathological state of infection, immune reaction and/or brain injury. These grouped changes are now being explored in more depth within this dataset, through additional proteomic studies of HIV infection, and using alterative assays with greater precision, including dedicated immunoassays and "targeted" LC-MS assays using multiple reaction monitoring MS [54-56].

While the relatedness of some of the identified proteins was obvious, reflecting the known linkages as part of the systemic host response to HIV infection and CNS injury, many were unknown or unexplored in this context. Thus, the protein abundance correlations provide new insight into the content and breadth of the changes in the CSF proteome over the course of HIV infected subjects. These correlations now need to be examined and dissected in more detail to define the underlying mechanistic pathways.

### Correlations of protein changes with orthogonal CSF biomarkers

Analysis of this varied patient cohort allowed for capturing a wide range of CNS states related to disease severity (Figure 1). Following initiation of cART a majority of patients (10/11) responded positively to therapeutic intervention as measured by reduction in HIV RNA, neopterin, and NFL levels, notably there was no reduction in the makers of disease for patient 4062 (Figure 1A). Because of the complexity of HIV infection in the CNS and its variable relationship to brain injury and the effects of therapy, classification of patients and their longitudinal samples can be difficult. One approach is the use of external biomarkers as guideposts for classification and interpretation of the proteomic changes. Each of the three external CSF biomarkers used in this analysis - HIV RNA, neopterin and NFL - provides a different view of CSF HIV infection and its consequences across a broad spectrum of progression (Additional file 1: Figure S1).

HIV RNA in CSF serves as an index of local infection, and directly traces the responses to antiretroviral therapy. However, the character and origins of CSF HIV are heterogeneous and can vary in genotype, receptor utilization, tropism, and resistance mutations [21,29,57-62]. Indeed, CSF HIV RNA represents the aggregate of contributions from more than one source, likely including both CD4+ T cells derived from blood and brain macrophages [20,62]. While the rates of response to therapy may vary, in general viruses of both types respond to therapy [5,63,64]. CSF HIV RNA provides a useful index to segregate samples for proteomic correlations, though is perhaps less useful for those with values below the level of detection (50 copies per mL) and does not include the HIV- subjects in which HIV was not measured. These omissions may contribute to the lower number of correlating proteins.

Neopterin, is a low-molecular mass pteridine metabolite of GTP that is produced largely by cells of the monocyte-macrophage lineage after activation, primarily by IFN-gamma [44]. Its concentration in CSF reflects local immune activation, a central aspect of HIV infection, and provides an index of progression across the entire disease spectrum, ranging from neuroasymptomatic infection with declining blood CD4+ T cell counts to HAD/HIVE, and with reduction in response to cART [25]. As expected, proteins that correlated with neopterin were predominantly associated with innate immune activation and inflammation processes. Given these considerations, it is perhaps not surprising that CSF neopterin provided the largest number of protein correlates. Additionally, CSF NFL was measured across the complete sample set.

CSF NFL can serve as an indicator of axonal injury in a wide variety of neurodegenerative conditions [65-72], including HIV infection [26]. It thus provides a direct indicator of active brain injury in the sample group. NFL provides an index of active injury, though in this study, this applied to only about 50 of the 92 samples, and thus does not provide information on infection or immune activation without this overt injury.

HIV encephalitis is sustained primarily in brain macrophages and is associated with widespread macrophage and microglial cells activation [73,74]. A simple model for the mechanism of neural damage includes both infection of these cells and their activation without infection leading to toxic events, including oxidative stress, that lead to neuronal and axonal damage [10,11]. In this simple model, it would be anticipated that NFL levels should correlate with those of neopterin and that both, in turn, would associate with perturbation of a similar set of brain proteins as disease develops and recovers following treatment. Surprisingly, we found two distinct populations of proteins correlating with neopterin and NFL, suggesting that pathogenetic linkages may be more complex.

The correlations within the proteome and with the three independent CSF biomarkers identified a number of proteins suitable for future exploration. For example procollagen C-proteinase enhancer-1 (gene symbol, PCPE-1) was negatively correlated (R = -0.29) with NFL and present at reduced levels (1.5 fold) in pretreatment HIV infected relative to post treatment samples. This finding is consistent with a previous report [35]. Protein PTPRZ1, receptor type tyrosine protein phosphatase zeta, has been demonstrated to be increased in expression in remyelinating oligodendrocytes associated with recovery and repair occurring in multiple sclerosis lesions [75] and was found to be positively correlated with NFL, possibly representing a compensatory measure to partially counter balance disease mediated axonal damage.

High levels of oxidative damage and oxidative stress have been linked to several neurodegenerative diseases including multiple sclerosis, Alzheimer's disease, Parkinson's disease, Huntington's disease, and amyotrophic lateral sclerosis [76]. Superoxide dismutase [Cu-Zn] 1 and 3 (gene symbols, SOD1 and SOD3) were identified in the present study and found to negatively correlate with neopterin. These results seemed surprising, since SOD3 expression is believed to be up regulated by IFN-γ and TNF-α together [77], while IFN-γ stimulates the production of neopterin. On the other hand, perhaps consistent with the present findings, SOD1 has been reported to be reduced in HIV-infected cognitively impaired patients [78]. The increase in SOD3 with treatment-related response may relate to CNS recovery mechanisms rather than injury.

### Defining pathogenetic pathways

Application of Ingenuity Pathway Analysis database suggests previously reported connections that may be involved in CNS HIV infection. Supporting the utility of this approach was the identification of APP as a "linking" node in the network identified among the neopterin correlations. A previous independent study showed that CSF concentrations of soluble APPs (both sAPP-alpha and beta) were reduced in ADC patients, and that this finding helped distinguish the CSF profile of ADC from that of Alzheimer's disease [27]. While the nature and cause of the reduction of APP remains uncertain, these findings justify further study of the amyloid pathway which has also been noted to be perturbed in other neuroinflammatory conditions [79]. Pathway analysis suggests other proteins could be pathogenetically linked to this pathway. Calsyntenin-1 (CLSTN-1) which was connected to APP in the network analysis (Figure 5A) and exhibited strong correlation with APP (R = 0.76) is expressed on neurons, participates with postsynaptic signaling, and has been reported to regulate APP cleavage. Kallikrein 6 (gene symbol, KLK6), a serine protease, was also linked to APP in the neopterin-related network. This protein plays a role in APP and α-synuclein degradation, potentially preventing aggregation of these proteins [80]. Further analysis of these and other associations and their linkages should provide a clearer and more comprehensive view of pathogenesis.

Autotaxin (gene symbol, ENPP2) has lysophospholipase D activity, converting lysophosphatidylcholine into lysophosphatidic acid (LPA) (reviewed in [81]) and signals through the LPA receptor, which is a g-protein coupled receptor and is highly expressed in the CNS [52]. In non-human primate studies a decrease in autotaxin was observed coincident with SIV infection [82] consistent with the current findings of reduced autotaxin expression with increased HIV disease severity. Supporting the nonhuman primate findings increased CSF levels of the substrate autotoxins enzymatic activity, lysophosphatidylcholine were reported in SIV-infected macaques [83]. Together these findings highlight the potential for extending 'omics measurements in biofluids such as CSF to include quantitative measurements of metabolites (metabolomics) and lipids (lipidomics). Parallel analysis of many 'omics measurements - what is broadly being defined as *panomics *- in CSF is likely to provide a rich and fruitful view of HIV-related CNS disease processes.

## Conclusions

Analysis of the CSF proteome of HIV infected individuals prior to and coincident with cART allowed the analysis of a wide range of disease severity in a relatively small number of samples. Proteome analysis has revealed many proteins exhibiting strong positive and negative correlation with CSF neopterin which serves as a broad marker of disease severity. This highlights the potential that these proteins may be participating in the ongoing CNS pathology and as such constitute targets of interest for development of better diagnostic, prognostic, and therapeutic tools to identify and manage ongoing disease related processes. Surprisingly, we found that different groups of proteins correlated with neopterin and NFL levels in the CSF, suggesting a decoupling of the processes associated with those once linked to pathological markers of poor patient prognosis in the absence of therapeutic intervention.

This exploratory study clearly shows the power and potential utility of CSF proteome analysis in providing a new and rich view of the effects of HIV infection on the CNS. Mass spectrometry-based proteome identification and label-free quantification of the CSF provides a wealth of data contributing to the characterization of CSF changes across the spectrum of disease and therapy. The findings now need to be explored in more depth as well as verified, validated, and extended with further studies. We are now pursuing these issues, and the lists of identified proteins in the supplementary tables should facilitate similar studies by other groups. The present studies of HIV also support a similar discovery approach to the study of other inflammatory and neurodegenerative conditions.

## Experimental procedures

### Study design and participants

CSF samples were selected from archived specimens obtained during two observational studies conducted at the University of California San Francisco (UCSF), one following the course of a cohort of HIV-infected subjects with periodic evaluations that included lumbar punctures (LPs), and the other study more directly evaluated CSF responses to initiation of cART [5,6]. The second study also included HIV seronegative (HIV-) volunteers (confirmed by serological testing) that served as controls for laboratory findings. These protocols were approved by the UCSF Committee on Human Research following federal guidelines. All CSF samples were obtained for study purposes with individual informed consent from each subject, and were accompanied by concurrent blood sampling along with general medical and neurological assessments as previously described [5,6,84] with the latter including assignment of ADC stage and brief quantitative neurological performance testing to derive an aggregate normalized Z score, the QNPZ-4 [85,86]. The overall strategy for this proteomic study involved two stages: an initial detailed analysis of four CSF samples (two each of HIV infected and uninfected controls) to define an AMT tag CSF database; and the main analysis, in which this database was applied to define peptides and proteins in the larger group of samples of interest.

### Fluid specimen processing and background laboratory studies

CSF total white blood cell (WBC) counts and differential, protein and albumin, along with blood albumin and CD4+ and CD8+ T lymphocyte counts by flow cytometry were performed in the San Francisco General Hospital (SFGH) Clinical Laboratories using routine methods in real time. For other CSF assays, including proteomics, CSF was first subjected to low-speed centrifugation to remove cells before aliquoting for storage at -80°C and later use.

HIV RNA was measured in cell-free CSF and plasma using the ultrasensitive Amplicor HIV Monitor assay (versions 1.0 and 1.5; Roche Molecular Diagnostic Systems, Branchburg, NJ) performed in the UCSF Virology Research Core Laboratory. We used a detection limit of 40 copies/mL and assigned a "floor" value of 39 copies/mL to lower values. HIV RNA levels were transformed to log_10 _values before analysis.

CSF neopterin concentrations were determined by enzyme-linked immunoassay in Innsbruck, Austria (BRAHMS GmbH, Hennigsdorf, Germany) [25]. CSF neurofilament light chain protein (NFL) concentrations were measured in the Clinical Neurochemistry Laboratory of the Sahlgrenska University Hospital in Gothenburg, Sweden using two methods, with the results of the second, newer, and more sensitive assay http://www.umandiagnostics.com/ "converted" to values comparable to the older method using linear regression to create a conversion formula. The aggregate results used the lower limit cutoff of 125 ng/mL from the older assay, and results below this concentration were expressed as 124 ng/mL for analysis [26,46].

### Sample preparation for proteomic analysis

Proteins in CSF samples were first denatured using 8 M urea, and disulfides were reduced following the addition of 5 mM dithiothreitol and incubating at 37°C for 60 min. Trypsin digestion was performed following diluting samples 4-fold with 50 mM ammonium bicarbonate (pH 8) and adding 1 mM CaCl_2_; the digestion reaction was carried out for 4 h at 37°C by addition of sequencing grade, modified porcine trypsin (Promega, Madison, WI) at a trypsin:protein ratio of 1:50, and then a second digestion reaction was performed after 2-fold dilution with the same buffer with the trypsin at a 1:50 trypsin/protein ratio at 37°C overnight. Tryptic peptide mixtures were cleaned by solid phase extraction using a 1-mL SPE C18 column (Discovery DSC-18, Supelco, Bellefonte, PA) as described previously [87]. Final peptide concentration was determined by BCA assay (Pierce). All tryptic digests were snap-frozen in liquid nitrogen and stored at -80°C until analysis.

Ammonium bicarbonate and acetonitrile were purchased from Fisher Scientific (Fair Lawn, NJ). Sequencing grade modified trypsin was purchased from Promega (Madison, WI). Bicinchoninic acid (BCA) assay reagents and standards were from Pierce (Rockford, IL). All other reagents were purchased from Sigma-Aldrich (St.Louis, MO). Water was purified using a Barnstead Nanopure Infinity water purification system (Dubuque, IA).

### Offline strong cation exchange fractionation

To develop the AMT tag database, tryptic peptide mixtures from CSF samples from two HIV- and two HIV + patients (see below for subject characteristics) were fractionated by strong cation exchange chromatography to reduce sample complexity. 150 μg of tryptic peptides per sample was resuspended in 900 μL 10 mM ammonium formate, pH 3.0; 25% acetonitrile and fractionated by strong cation exchange chromatography as described previously [87]. 15 fractions were collected for each sample and lyophilized prior to high resolution, reversed phase LC-MS/MS analysis.

### Reversed-phase capillary LC-MS/MS and LC-MS analysis

For analysis of the main sample group, peptide samples were separated by high resolution, reversed phase liquid chromatography (LC) as previously described using a four-column capillary LC system [88] in-line with the mass spectrometer (MS). Strong cation exchange fractions were analyzed using an LTQ mass spectrometer (ThermoFinnigan, San Jose, CA) operated in data-dependent mode (m/z 400-2000) with the top 10 most intense ions being isolated. For label-free quantification using unfractionated individual CSF samples, an Exactive mass spectrometer (ThermoFinnigan, San Jose, CA) obtained full scan MS spectra (m/z 400-2000) with resolution of > 30,000 at m/z 400 (accumulation target: 1,000,000).

LC-LTQ MS/MS raw data was extracted using Extract_MSn (version 3.0) and analyzed with the SEQUEST algorithm (V27 revision 12) searching the MS/MS data against the human IPI database which contained 69,731 total protein entries (Version 3.39, released February 7, 2008). Precursor mass tolerance of 3 Daltons and 1 Dalton for MS/MS ion masses without an enzyme defined were used for search parameters. The LTQ MS/MS data were processed by in-house software DeconMSn [89] accurately determining the monoisotopic mass and charge state of parent ions, followed by SEQUEST search against the IPI database in the same fashion as described above. Data filtering criteria based on the cross correlation score (Xcorr) and delta correlation (ΔCn) values along with tryptic cleavage and charge states were developed using the decoy database approach and applied for filtering the raw data to limit false positive identifications at the peptide level [90-92]. All peptides identified had a peptide prophet probability equal to or greater than 0.5 and Xcorr > = 1.9, 2.2, or 3.5 for 1+, 2+, or > = 3+ if seen in one MS/MS spectrum or an Xcorr > = 1.9 if observed in 2 or more MS/MS spectra.

The accurate mass and time (AMT) tag strategy [43] was used for label-free quantification of MS features observed in the Exactive MS analysis of the individual CSF samples from HIV- and HIV + sample sets. The filtered MS/MS peptide identifications obtained from the 2D-LC-MS/MS analyses of all pooled CSF samples were included in an AMT tag database with their theoretical mass and normalized elution time (NET; from 0 to 1) recorded. LC-MS datasets were then analyzed by in-house software VIPER [93] that detects features in mass-NET space and assigned them to peptides in the AMT tag database [33]. The distribution of mass deviation (from the theoretical masses) was determined as having a standard deviation (σ) of 2.024 part per million (ppm), and identified MS features with mass error less than +/- 10 ppm were used for inferring protein level abundance. The resulting lists of peptides from the direct LC-MS analysis were further analyzed by Protein Prophet software [94] to remove redundancy in protein identification.

### Data analysis

To determine quantitative changes in protein abundances between control HIV-uninfected and HIV-infected subjects' CSF samples, we used in-house developed software DAnTE [95]. Briefly, peptide intensities from the LC-MS analyses were log2 transformed. Peptide abundances were then "rolled up" to the protein level employing the R-rollup method (based on observed peptide level trends) implemented in DAnTE [41]. All proteins were quantified by considering ion intensities of a minimum of 2 peptides. ANOVA and clustering analyses were also performed using DAnTE. Sample and protein abundance correlation analysis was performed using the statistical software package R http://www.R-project.org.

Determination of functional enrichment of proteins clustered together by cross correlation was performed employing Gominer [47]. Network analysis was performed through the use of Ingenuity Pathways Analysis (Ingenuity^® ^Systems, http://www.ingenuity.com). The functional analysis identified the biological functions and/or diseases that were most significant to the dataset. Molecules from the dataset that met the cross correlation filter cutoff of R ≤ -0.3 or ≥ 0.3 and a P value of ≤ 0.05 by *t*-test (Pearson) and were associated with biological functions and/or diseases in Ingenuity's Knowledge Base were considered for the analysis. Right-tailed Fisher's exact test was used to calculate P-values determining the probability that each biological function and/or disease assigned to that dataset was due to chance alone. Correlations of the CSF marker findings across the sample set used Spearman's non-parametric correlation.

## Competing interests

The authors declare that they have no competing interests.

## Authors' contributions

TEA performed data analysis, interpretation and manuscript preparation. JMJ participated in study design, data analysis, interpretation, and manuscript preparation. SSS participated in study design, sample acquisition, data analysis, interpretation and manuscript preparation. MAG participated in sample preparation and data acquisition and manuscript preparation. DF participated in data acquisition, analysis, and manuscript preparation. TL participated in sample acquisition and preparation. HZ participated in data acquisition, data analysis, and manuscript preparation. DGC participated study design, data interpretation, and manuscript preparation. RWP participated in study design, sample acquisition, data analysis, interpretation, and manuscript preparation. RDS participated in study design, data analysis, interpretation, and manuscript preparation. All authors read and approved the final manuscript.

## Supplementary Material

Additional file 1 **Figure S1**. Comparison of concentrations of three orthogonal CSF biomarkers across the sample set. In order to visually compare the range of concentrations of the three CSF biomarkers across the entire sample sets, they were independently ordered from highest to lowest and this ranking was then applied to the other two biomarkers and displayed on the same row. The top row shows results ranked by CSF HIV RNA concentration for these values (left), CSF neopterin (middle) and CSF NFL (right). Both visually and by Spearman's rank (P values and correlation coefficient shown within individual figures) it can be seen that CSF neopterin values correlate more closely with HIV RNA (r = 0.612) than do CSF NFL values (r = 0.289). Also note that the low CSF HIV RNA values are at the limit of detection (dotted line) and that this was not measured in the HIV- subjects. The second row ranks results by CSF neopterin. In addition to the correlation with CSF HIV RNA (right), it also shows that the CSF NFL values are less well correlated (r = 0.235) and that many high NFL values were noted in patients with lower CSF neopterin. The bottom row shows ranking by CSF NFL and again emphasizes that elevated CSF HIV RNA and neopterin was noted even in the subjects with CSF NFL below the level of detection (dotted line in lower right graph). Click here for file

Additional file 2 **Tables S1**. Peptides identified in the cerebrospinal fluid. Click here for file

Additional file 3 **Tables S2**. Proteins identified in the cerebrospinal fluid of subject samples and correlation analysis of protein abundance with orthogonal markers of HIV CNS infection and pathology. Click here for file
