# Supplementary figures and images for: The cerebrospinal fluid proteome in HIV infection: change associated with disease severity
Source: Clin Proteomics. 2012 Mar 20;9(1):3. doi: 10.1186/1559-0275-9-3 (PMC3353874; doi:10.1186/1559-0275-9-3)

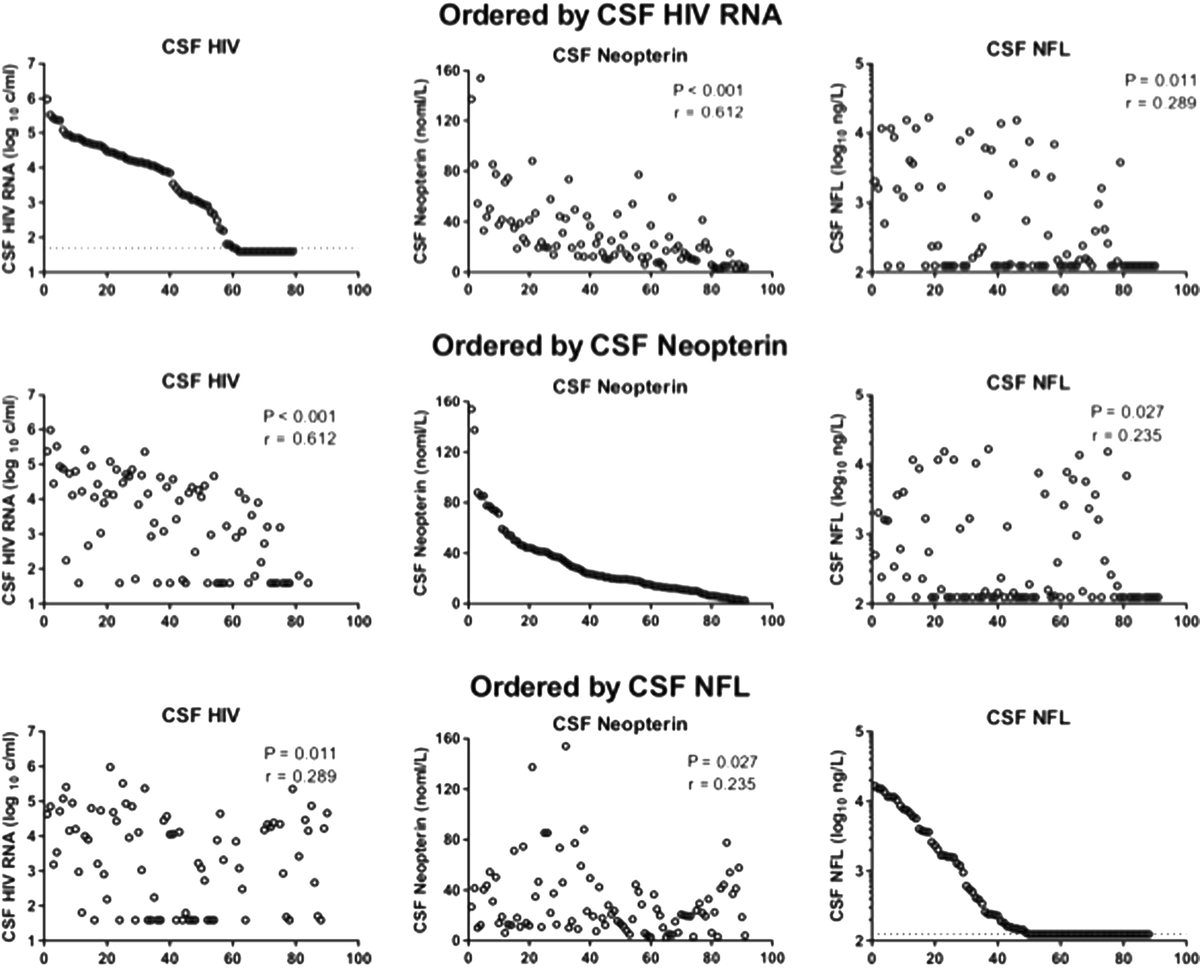

Supplement: Additional file 1 — Figure S1. Comparison of concentrations of three orthogonal CSF biomarkers across the sample set. In order to visually compare the range of concentrations of the three CSF biomarkers across the entire sample sets, they were independently ordered from highest to lowest and this ranking was then applied to the other two biomarkers and displayed on the same row. The top row shows results ranked by CSF HIV RNA concentration for these values (left), CSF neopterin (middle) and CSF NFL (right). Both visually and by Spearman's rank (P values and correlation coefficient shown within individual figures) it can be seen that CSF neopterin values correlate more closely with HIV RNA (r = 0.612) than do CSF NFL values (r = 0.289). Also note that the low CSF HIV RNA values are at the limit of detection (dotted line) and that this was not measured in the HIV- subjects. The second row ranks results by CSF neopterin. In addition to the correlation with CSF HIV RNA (right), it also shows that the CSF NFL values are less well correlated (r = 0.235) and that many high NFL values were noted in patients with lower CSF neopterin. The bottom row shows ranking by CSF NFL and again emphasizes that elevated CSF HIV RNA and neopterin was noted even in the subjects with CSF NFL below the level of detection (dotted line in lower right graph). [file 1559-0275-9-3-S1.JPEG]
